# Supplementary material for: MetaQTL: a package of new computational methods for the meta-analysis of QTL mapping experiments
Source: BMC Bioinformatics. 2007 Feb 8;8:49. doi: 10.1186/1471-2105-8-49 (PMC1808479; doi:10.1186/1471-2105-8-49)
Supplement: Additional File 7 — MetaQTL Package : jar file and tutorial. This Zip archive contains both the MetaQTL JAR file and the files of the tutorial. [file 1471-2105-8-49-S7.zip › org.inra.metaqtl/doc/org/thalia/bio/entity/bean/class-use/BioEntityBean.html]

Uses of Class org.thalia.bio.entity.bean.BioEntityBean


|  |  |  |
| --- | --- | --- |
| **Overview** \| **Package** \| **Class** \| **Use** \| **Tree** \| **Deprecated** \| **Index** \| **Help** | |  |
| PREV   NEXT | **FRAMES**    **NO FRAMES**     **All Classes** |  |


---


## **Uses of Class org.thalia.bio.entity.bean.BioEntityBean**

| Packages that use BioEntityBean | |
| --- | --- |
| **org.thalia.bio.entity.adapter** |  |
| **org.thalia.bio.entity.bean** |  |

Uses of BioEntityBean in org.thalia.bio.entity.adapter

| Methods in org.thalia.bio.entity.adapter with parameters of type BioEntityBean | |
| --- | --- |
| `static void` | `BioEntityAdapter.fromEntity(IBioEntity entity, BioEntityBean bean)` |
| `static void` | `BioEntityAdapter.toEntity(BioEntityBean bean, IBioEntity entity)` |

Uses of BioEntityBean in org.thalia.bio.entity.bean

| Subclasses of BioEntityBean in org.thalia.bio.entity.bean | |
| --- | --- |
| `class` | `BioEntityCollectionBean`             Class Description Here |
| `class` | `GeneticMapBean`             Class Description Here |
| `class` | `LGroupBean`             Class Description Here |
| `class` | `LocusBean`             Class Description Here |
| `class` | `OntologyBean` |
| `class` | `OntologyTermBean` |

---


|  |  |  |
| --- | --- | --- |
| **Overview** \| **Package** \| **Class** \| **Use** \| **Tree** \| **Deprecated** \| **Index** \| **Help** | |  |
| PREV   NEXT | **FRAMES**    **NO FRAMES**     **All Classes** |  |


---
